# Probable awake bruxism - prevalence and associated factors: a cross-sectional study

**DOI:** 10.1590/2177-6709.27.4.e2220298.oar

**Published:** 2022-08-15

**Authors:** Priscila Brenner HILGENBERG-SYDNEY, Ana Laura LORENZON, Giovanna PIMENTEL, Ricardo Rasmussen PETTERLE, Daniel BONOTTO

**Affiliations:** 1 Universidade Federal do Paraná, Faculdade de Odontologia (Curitiba/PR, Brazil).; 2 Universidade Federal do Paraná, Faculdade de Medicina (Curitiba/PR, Brazil).

**Keywords:** Anxiety, Bruxism, Teeth grinding disorder, Temporomandibular joint disorders

## Abstract

**Introduction:**

Bruxism is defined as a repetitive activity of masticatory muscles, characterized by the clenching or grinding of the teeth, which can occur during wakefulness (awake bruxism) or during sleep (sleep bruxism).

**Objectives:**

The objectives of the present study were to determine the prevalence of awake bruxism and its associated factors.

**Methods:**

Sample was composed by 50 participants of both genders, aged between 18 and 60 years, submitted to a clinical examination - to observe the presence of tooth wear, marks on the mucosa, or masseter muscles hypertrophy - and self-applied questionnaires, which evaluated the presence of TMD signs and symptoms, oral behaviors, lifestyles, anxiety level and sleep quality.

**Results:**

The prevalence of awake bruxism was 48%. Its presence was statistically and significantly associated with the presence of signs and symptoms of TMD (*p*=* *0.002), poor sleep quality (p* *=* *0.032), buccal mucosa indentations (p* *<* *0.001) and tongue (p* *=* *0.011). Age, gender, social characteristics, habits (such as coffee ingestion, smoking, alcoholism and physical activity) and tooth wear were variables that had no significant association with awake bruxism.

**Conclusions:**

It was concluded that awake bruxism shows a high prevalence and a positive association with signs and symptoms of TMD and worst sleep quality. In addition, awake bruxism is more likely to occur in individuals who have buccal mucosa indentation and who present high rates of oral habits and oral behaviors.

## RESUMO

**Introdução:**

O bruxismo é definido como a atividade repetitiva dos músculos mastigatórios, e pode ocorrer durante o período acordado (bruxismo em vigília) ou durante o sono (bruxismo do sono).

**Objetivo:**

O presente estudo teve como objetivo avaliar a prevalência de bruxismo em vigília e seus fatores associados.

**Métodos:**

A amostra foi composta por 50 participantes, de ambos os sexos, com idades de 18 a 60 anos, avaliados por meio de minucioso exame clínico - para observar se havia presença de sinais como desgaste dentário, indentações na mucosa, hipertrofia do músculo masseter - e de questionários autoaplicáveis que visaram avaliar a presença de sinais e sintomas de disfunção temporomandibular (DTM), os comportamentos orais e hábitos de vida, o nível de ansiedade e a qualidade de sono dos participantes.

**Resultados:**

A prevalência de bruxismo em vigília foi de 48%. Sua presença foi associada, estatística e significativamente, com a presença de sinais e sintomas de DTM (*p*=* *0,002), má qualidade do sono (*p*=* *0,032), e indentações na mucosa jugal (*p*<* *0,001) e língua (*p*=* *0,011). Por outro lado, a idade, sexo, características sociais, hábitos (como ingestão de café, fumo, álcool e prática de atividade física), e o desgaste dentário foram variáveis que não tiveram associação com o bruxismo em vigília.

**Conclusão:**

Diante disso, conclui-se que o bruxismo em vigília possui uma prevalência significativa e uma associação positiva com DTM e qualidade do sono. Além disso, o bruxismo em vigília apresenta maior probabilidade de acontecer em indivíduos com indentação de mucosa jugal e com alta prevalência de hábitos e comportamentos orais.

## INTRODUCTION

Bruxism is a broad spectrum of masticatory muscles activity, characterized by grinding/clenching of the teeth and/or clenching with increased thrust and mandibular tonus. It can happen in two different moments, during sleep (sleep bruxism) or during wakefulness (awake bruxism).^1^ ^,^ ^2^ It is a very common condition in the general population (80-90%), in which at some point in life people will tighten or grind their teeth to some degree.^3^

Bruxism has possible consequences on the different components of the stomatognathic system (teeth, periodontium, masticatory muscles and temporomandibular joint).^4^ Therefore, the concern of dental specialists who work with Oral Rehabilitation and Orthodontics about its characteristics, diagnosis and influence during dental treatment.

There are many differences between awake bruxism and sleep bruxism.^5^ Awake bruxism (AB) is usually a semi-voluntary activity of clenching of the teeth that does not produce sound, and the individual is aware of this condition. During sleep, episodes of bruxism can be accompanied by the unconscious grinding of the teeth, in which the person will only acknowledge this activity through the reports of a bed partner or roommate.^3^ ^,^ ^6^

The etiology of bruxism is a complex and controversial process. In the past, a peripheral etiology for sleep and awake bruxism was suggested, in which occlusal interferences would be able to stimulate and trigger non-functional masticatory movements. However, after some years of research, it was necessary to separate awake bruxism and sleep bruxism as separate entities, once they seem to have different characteristics, etiology and associated factors. Currently, the most plausible hypothesis admits a multifactorial model for the etiology of bruxism, in which psychosocial and pathophysiological aspects interact with morphological-peripheral characteristics.^5^

Psychosocial factors such as stress, anxiety, and certain personality traits are often mentioned associated to awake bruxism and sleep bruxism.^7^ ^,^ ^8^ Thus, several studies point to the fact that awake bruxism and sleep bruxism should be considered two different disorders, due to their distinct etiology and pathophysiology.^5^

According to a study^6^ that investigated the association between bruxism and painful temporomandibular disorders (TMD) in adults, the presence of awake bruxism or sleep bruxism increases the risk for development of pain, suggesting a possible interaction between these conditions. Thus, the aim of the present study was to evaluate the prevalence of awake bruxism and its association with signs and symptoms of TMD, oral habits, anxiety level and sleep quality. 

## MATERIAL AND METHODS

Patients who sought care in the Dental Clinics of *Universidade Federal do Paraná* (UFPR), from February 2019 to May 2019, were verbally invited to participate in this study. Individuals of both genders were included, aged between 18 and 60 years. To participate, all patients had to read and agree with an Informed Consent Term, approved by the local Research Ethics Committee, under protocol #1,705,268.

Sample size estimation to evaluate the prevalence of AB in the studied population was performed considering the following parameters: population of 61 individuals in the waiting list for general dental screening evaluation, with no main chief complaint, an anticipated frequency of 50%, and a confidence limit of 5%. Using these parameters, the calculations resulted in a sample size of 53 individuals. Patients with ongoing pain, or that were specifically looking for treatment of orofacial pain and/or bruxism belonged to other waiting lists, that were not considered in the present study.

All participants were interviewed and evaluated by two experimented researchers, and a pilot study was previously conducted for calibration. To minimize discrepancies in the interpretation of the diagnoses, calibration was done by comparing examinations of the same participant by different examiners, and by having the examiner perform the same examination at different times. The intraexaminer agreement was k = 0.877 (*p*≤ 0.001), and k = 0.889 (*p*≤ 0.001) for examiners one and two, showing an almost perfect agreement. The interexaminers Kappa agreement coefficient (k = 0.794) (*p*≤ 0.001) showed a strong/substantial agreement between examiners. Participants answered general screening questions such as age, gender, marital status, occupation, habits and lifestyle. Afterwards, the participants underwent a clinical examination, which aimed to detect the presence of clinical signs such as tooth wear facets, indentation on tongue, lips and oral mucosa, and also muscular hypertrophy, by digital palpation of the masseter muscles. After clinical examination, participants were asked to fill out four specific self-applied questionnaires:

- *TMD Screening Questionnaire proposed by the American Academy of Orofacial Pain (AAOP)*: This questionnaire is composed by 10 objective questions that aim to evaluate the following items: pain and/or mouth opening limitation; joint sounds; pain in the temples, cheeks or periauricular region; headaches; neck pain; toothache; history of trauma to the head, neck or mandible; history of recent change in occlusion; history of recent temporomandibular joint locking. The TMD screening questionnaire was used to verify the presence of TMD signs and symptoms. If the participant answers “yes” on at least one of the questions, it is already an indication for a specialist evaluation.^9^
- *Oral Behaviors Checklist (OBC)*: Composed by 21 objective questions, this questionnaire was applied in order to verify oral behaviors of the participants. The total score may vary from 0 to 84, and is obtained by the sum of the responses. Each answer option corresponds to a score from 0 to 4, as follows: “no time” = 0; “small part of the time” = 1; “some part of the time” = 2; “most of the time” = 3 and “all times”= 4. The higher the total score, the higher the rate of oral behavior.^10^
- *State-Trait Anxiety Inventory (STAI)*: It consists of a two-part self-applied questionnaire. The first one evaluates anxiety “state” and the second, a “trace” of anxiety in the participant. This questionnaire was created^11^ and validated for the Brazilian Portuguese.^12^ In the present study, only the first part of the questionnaire was applied, which gave us an anxiety “state” score. There were 20 items with statements, the answers of which corresponded to a score that ranged from 0 to 80. The possible answers were: “no” = 1, “a little” = 2, “quite” = 3 or “totally” = 4. The higher the score obtained, the greater the level of anxiety of the patient. The weights of the items are equal to the scored points (from 1 to 4) marked by the patient, and the items in which the high score indicates high anxiety level remain with this same weight; but the items in which the high scores indicate low anxiety level have their weights inverted, thus avoiding tendency of response.
- *Pittsburgh Sleep Quality Index (PSQI-BR)*: Composed by objective questions, this questionnaire aims to evaluate the quality of sleep. The sleep-related components evaluated were: subjective sleep quality; time to fall asleep; sleep duration; habitual sleep efficiency; sleep disorders; use of sleeping medication and daytime dysfunction. Each of the components receives a score that ranges from 0 to 3. All scores are summed up, ranging from 0 to 21. General population score reaches score 5. From that on, the higher the score, the worse the quality of sleep.^13^

For awake bruxism diagnosis, a classification system suggested by an international group of experts was adopted, grading the condition into “possible”, “probable” and “definitive”. According to this group of authors, “possible” bruxism is based only on positive self-report; “probable” is based on specific clinical examination, with or without self-report; and “definitive” is based on the self-report, specific instrumental assessment, with or without self-report and/or positive clinical examination.^1^ In the present study, participants were graded according to “probable” awake bruxism (PAB).

Data were gathered and analyzed using R statistical software (version: 3.4.0.), applying Fisher’s Exact, Mann-Whitney and Logistic Regression tests, always maintaining a significance level of 5%.

## RESULTS

From the 53 patients recruited to be included, 3 declined to participate. The sample was comprised of 50 participants, 37 (74%) were women and 13 (26%), men; with a mean age of 33.0±12.5 years, ranging from 18 to 58. The mean age difference between women (31.29* *±* *11.47 years) and men (38.0* *±* *14.27 years) was not statistically significant (*p*=* *0.182). Table 1 shows the association between mean age and probable awake bruxism (PAB), which was not statistically significant. Also, it describes the association between PAB and gender, and also the total prevalence of patients with and without PAB.

**Table 1:** Descriptive table of the patients with/without PAB.

	With PAB	Without PAB	p
Age	31.87 (±11.91)	34.11 (±13.09)	0.476
Female (n)	20	17	0.202
Male (n)	4	9	
Total n (%)	24 (48%)	26 (52%)	

PAB = probable awake bruxism; * statistically significant.

**Table 2:** Association between clinical signs and PAB.

Clinical sign	p
Masseter hypertrophy	0.190
Tongue indentations	0.011*
Labial mucosa indentations	0.480
Buccal mucosa indentations	<0.001*
Tooth wear	0.225

PAB= probable awake bruxism; * statistically significant.

Clinical examination was performed to detect some signs of AB. PAB was significantly associated with the presence of tongue and buccal mucosa indentations. Results are detailed in Table 2. 

The association between PAB and the presence of TMD signs and symptoms was significant (*p*=* *0.002). Among the 24 patients diagnosed with PAB, 21 presented signs and/or symptoms of TMD. As for the 26 patients without PAB, 12 presented signs and/or symptoms of TMD. 

Table 3 shows the average scores from the specific questionnaires applied. The OBC and PSQI-BR results presented average scores significantly higher for individuals with PAB, compared to those without PAB. In addition, the anxiety questionnaire (STAI) presented higher average scores for individuals with PAB, even though the difference was not statistically significant. There was a statistically significant association between the results of the OBC scores and the anxiety state (STAI) for patients diagnosed with PAB (*p*=* *0.01). For the patients without PAB, no association was found (*p*=* *0.38). Also, there was a positive association (*p*=* *0.004) between the OBC score and the presence of TMD signs and symptoms in the evaluated sample.

**Table 3:** Scores obtained from the STAI, OBC and PSQI-BR questionnaires for patients with and without PAB, including mean and standard deviation.

	With PAB (n=24)	Without PAB (n=26)	p
OBC	43.08 (±10.00)	38.84 (±6.44)	0.251
STAI	27.20 (±11.15)	13.61 (±8.96)	< 0.001*
PSQI-BR	7.87 (±4.01)	5.46 (±2.58)	0.032*

PAB: probable awake bruxism; SD: standard deviation; STAI: State-Trait Anxiety Inventory; OBC: Oral Behaviors Checklist; Pittsburgh Sleep Quality Index (PSQI-BR); * statistically significant.

Regarding socioeconomic characteristics, there was no statistically significant association between PAB and occupation (*p*=* *0.19), marital status (*p*=* *0.51) and parenthood (*p*=* *0.57) of the individuals evaluated.

**Figure 1:**
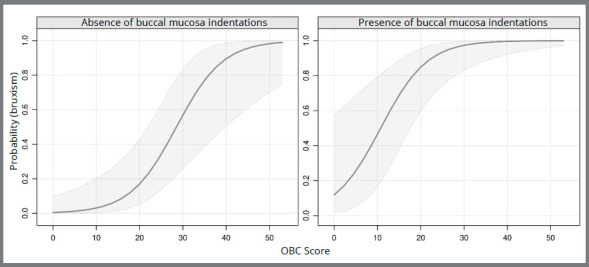
Probability of PAB diagnosis based on the OBC score in individuals with and without buccal mucosa indentations. OBC: Oral Behaviors Checklist; PAB: probable awake bruxism.

As observed, several significant variables were found in association with the presence of PAB, such as: OBC and sleep quality (PSQI-BR), presence of TMD signs and/or symptoms, presence of tongue and buccal mucosa indentations. After performing the stepwise procedure, the following covariables remained: OBC score and the presence of buccal mucosa indentations (*p*<* *0.001). The probabilities were calculated through Logistic Regression, and the analysis graphics are shown in Figure 1. 

At first, these graphics may seem very similar, but a careful observation reveals interesting data about the probability of a patient with or without buccal mucosa indentation to present PAB diagnosis, based on the OBC score. An individual with a score of approximately 20 points on the OBC who does not present buccal mucosa indentations, demonstrates a probability of approximately 0.2 for PAB. While an individual with the same score on the OBC, but with the presence of buccal mucosa indentations, shows a probability higher than 0.8 for PAB.

There were some questions concerning lifestyle habits, but none presented statistically significant association with the diagnosis of PAB, namely: physical activity practice (*p*= 0.78), smoking (*p*= 1.00), ingestion of alcohol (*p*= 0.46) and caffeine (*p*= 0.75). The dental history of the patients was also assessed, but there were no positive associations between PAB and fractured teeth (*p*= 0.53), orthodontic treatment (*p*= 0.57), nor previous treatment for bruxism (*p*= 0.23).

## DISCUSSION

The study of awake bruxism is a more recent topic in the literature, comparing to sleep bruxism. This study may contribute in part to a better understanding of the differences between the types of bruxism and its associated factors. Some authors have suggested that bruxism seems to be a risk factor for some people, and a protective factor for others.^1^ ^,^ ^2^ Among the 50 participants in the present study, 24 (48%) presented probable awake bruxism (PAB), and there was a statistically significant association with factors such as TMD signs and/or symptoms, sleep quality and other oral behaviors/habits. 

One of the objectives of this research was to evaluate the prevalence of “probable” awake bruxism (PAB), which was of 48%. According to a literature review,^14^ awake bruxism has a prevalence of 22.1% to 31%. The divergence with the present results can be explained by two aspects. The first one refers to the location where the participants were selected: As the present study was performed in a dental care referral center inside the same university (UFPR), it may have caused a bias in sample selection. Another aspect that must be considered is the differences in the diagnostic criteria adopted for each study, which represents a recognized difficulty in the field of bruxism, since there is still no established standardization for comparison of results. 

Regarding the gender of the participants, although female rate was higher, there was no significant association between gender and PAB. This result is in agreement with previous studies^15^ ^,^ ^16^ in which this association was not positive either. Patients with PAB presented a mean age of 31.87 years, and patients without PAB, 34.11 years; as for the association between mean age and awake bruxism, there was no statistical significance. This result was also found in another study,^16^ in which the difference between the mean age for patients with and without any type of bruxism was not significant. This study has also used self-report and clinical examination as diagnostic criteria for characterizing probable awake bruxism.

The clinical examination performed in the present research showed statistically significant results for marks/indentations in tongue and oral mucosa of AB patients. These marks were frequently present in AB individuals, however it should be bear in mind that they cannot be considered exclusive of AB. Other oral habits such as nail biting and digital sucking can also leave those marks. On the other hand, there was no statistically significant association with tooth wear. In the literature, several studies indicate tooth wear as a way of assessing the presence of bruxism, but recently it has been described as a poor indicator, besides the fact that it does not discriminate awake bruxism from sleep bruxism.^7^ Many patients may present tooth wear as a “scar” caused by another reason or even by a bruxism period experienced in the past. Besides that, bruxism can be present without tooth contact, only with muscle contractions and/or thrusting the mandible, so tooth wear may not be present even with active AB.^1^

Temporomandibular disorders comprise a variety of conditions that affect temporomandibular joint (TMJ) and/or masticatory muscles, and is characterized by a multifactorial etiology, following a biopsychosocial model.^17^ Among the various etiologies of TMD it is known that parafunctional habits, especially awake bruxism, are predisposing, initiating and/or perpetuating factors.^9^ In addition, the results of the present study demonstrated a significant association between oral behaviors and TMD signs and symptoms (*p*= 0.004). Besides that, the presence of TMD signs and symptoms in individuals with PAB was statistically significant, in agreement with the majority of authors. 

Another study^18^ investigated the correlation between self-reported and clinical examination based on diagnoses of awake bruxism and sleep bruxism. The sample was comprised of TMD patients. An expressive correlation was found between self-reported and clinical diagnosis for awake bruxism, in which 50.6% of the patients reported presenting this type of bruxism, while in the clinical examination the diagnosis was detected in 52.5% of the patients. Besides this correlation, awake bruxism was also associated with TMD signs and symptoms. In the present study, 24 of the 33 individuals with TMD signs and symptoms also presented PAB, demonstrating a statistically significant association (*p*=* *0.002). 

Previous authors^19^ aimed to compare the prevalence of cervical muscle pain and TMD among dentists and other professionals, and a positive association between myogenic TMD and sleep bruxism and awake bruxism was found. Also, in another study,^6^ the association between TMD pain with awake bruxism and sleep bruxism in adults was evaluated. The results showed that the risk of such pain increases significantly in individuals with the two types of bruxism. A case-control^20^ study evaluated patients with painful TMD, considered as case group, and pain-free or absence of TMD patients, considered the control group. This study aimed to investigate the associations between both awake bruxism and sleep bruxism with painful TMD. 

As for the results, bruxism was reported most in case rather than control group, indicating that both types of bruxism are considerably associated with painful TMD. The present study is in accordance with the recent literature, indicating that TMD signs and symptoms and awake bruxism are associated. However, causality cannot be established between the two conditions.

The OBC results demonstrated a significant association with the diagnosis of PAB. In a study conducted by other authors,^10^ the same questionnaire was applied in order to verify positive association between oral behaviors and psychosocial factors such as stress, depression and anxiety. All these associations were significant, which is in agreement with the present study.

The results show that an individual that presents marks/indentations on buccal mucosa is more likely to possess PAB, based on the score obtained in the OBC. In addition, the higher the index of oral habits, the greater the likelihood of the patient developing awake bruxism. This is particularly valuable to be used for comparison in dental practice, as follows: the clinician may apply the OBC before and during treatment, to monitor the presence of oral habits and awake bruxism, and therefore prevent or manage its consequences. 

Psychosocial factors such as anxiety and stress may be related to the etiology of bruxism.^7^ The association between anxiety level obtained through the STAI questionnaire and PAB was not statistically significant. However, it can be observed that patients with PAB presented higher scores than those without the condition. Therefore, these values might be considered clinically relevant, since each group fit in a different anxiety state: “medium” and “low”, respectively. This classification is in agreement with another sutdy,^21^ which divided the scores 20 to 40 in “low” anxiety, 40 to 60 in “average” anxiety and 60 to 80 in “high” anxiety. Besides that, the possibility of bias in the sample selection of the present study should be mentioned, since the individuals who were evaluated were waiting for dental screening at the same university (UFPR), which means they could have their anxiety state altered at that specific moment, regardless of the PAB diagnosis. Another possible explanation for the lack of statistical significance is the relatively small size of the studied sample.

Differently from the present results, some studies in the literature found significance between emotional factors and PAB. A literature review by experts^5^ evaluated the association between psychosocial factors and bruxism, adopting as diagnostic criteria polysomnography, self-report and/or clinical examination. A positive association of bruxism with stress was reported by some studies that adopted self-reported as diagnostic method. Some other authors^16^ evaluated the prevalence of psychopathological symptoms in patients who self-reported different forms of bruxism. In their study the participants had to complete the SCL-90-R questionnaire for psycho-pathological aspects evaluation, and on average patients with awake bruxism had higher scores than those with sleep bruxism or absence of bruxism. 

Poor quality of sleep is also a factor that seems to be related to PAB, as shown in the results of the present study. Another group of authors^22^ evaluated the association between sleep bruxism, awake bruxism and quality of sleep among students of Dentistry. The Brazilian version of the Pittsburgh Sleep Questionnaire Index (PSQI-BR) was applied to a sample composed by 183 students. In addition, these students answered two questions related to awake bruxism and sleep bruxism, qualified by means of self-report, with a prevalence of 21.5% and 36.5%, respectively. Poor sleep quality was a statistically significant factor in association with the two types of bruxism.

Some habits such as smoking and consumption of caffeine and/or alcohol were also questioned to the participants, which showed no positive association with awake bruxism. This result is corroborated by another study^23^ based on the habits of a group of college students, in which there was no significant association between coffee/tobacco consumption and awake bruxism. On the other hand, a systematic review of the literaure^24^ indicated that sleep bruxism appears to be associated with alcohol, caffeine and tobacco use. This divergence in results might be explained by the fact that their focus was sleep bruxism, while ours was PAB, which are conditions with clear different etiology and pathophysiology.^5^

Some limitations of the present study are that many of the previous investigations regarding AB have solely diagnosed it by self-report. The present study is one of the few studies that evaluated the clinical signs, in order to get a more reliable AB diagnosis. Although, it still is not the gold standard for AB diagnosis, according to experts.^2^ The definite awake bruxism diagnosis should include the ecological momentary assessment,^2^ which was not performed in this investigation. This was a screening study with a limited sample size, which cannot be considered representative of the general population nor the patients population. As for mean age, which ranged from 18 to 58 years, a larger sample size would be better to allow a statistical analysis by age groups. In addition, due to the greater number of women in the sample and some old enough to be in menopause,^25^ the results of the anxiety and quality of sleep may also have suffered some type of bias. Finally, divergence in the results shows that in fact awake bruxism is related to several factors, proving its multifactorial etiology. The lack of studies involving awake bruxism justifies the need for further research in this area, in order to obtain results that may be compared, thus contributing to a better understanding of its pathophysiology and, consequently, adequate therapeutic approach.

## CONCLUSIONS

The prevalence of probable awake bruxism was 48%. The presence of buccal mucosa indentation enhances the probability of PAB. Also, PAB is associated with signs and symptoms of TMD, oral habits and poor sleep quality. 
